# A plasma miRNA-based classifier for small cell lung cancer diagnosis

**DOI:** 10.3389/fonc.2023.1255527

**Published:** 2023-10-05

**Authors:** Michela Saviana, Giulia Romano, Joseph McElroy, Giovanni Nigita, Rosario Distefano, Robin Toft, Federica Calore, Patricia Le, Daniel Del Valle Morales, Sarah Atmajoana, Stephen Deppen, Kai Wang, L. James Lee, Mario Acunzo, Patrick Nana-Sinkam

**Affiliations:** ^1^ Department of Internal Medicine, Division of Pulmonary Diseases and Critical Care Medicine, Virginia Commonwealth University, Richmond, VA, United States; ^2^ Department of Molecular Medicine, University La Sapienza, Rome, Italy; ^3^ Center for Biostatistics, The Ohio State University, Columbus, OH, United States; ^4^ Department of Cancer Biology and Genetics, The Ohio State University, Columbus, OH, United States; ^5^ Vanderbilt University Medical Center and Tennessee Valley Healthcare System, Nashville, TN, United States; ^6^ Institute for System Biology, Seattle, WA, United States; ^7^ Department of Chemical and Biomolecular Engineering, The Ohio State University, Columbus, OH, United States

**Keywords:** small cell lung cancer, microRNAs, biomarkers, oncology, classifier

## Abstract

**Introduction:**

Small cell lung cancer (SCLC) is characterized by poor prognosis and challenging diagnosis. Screening in high-risk smokers results in a reduction in lung cancer mortality, however, screening efforts are primarily focused on non-small cell lung cancer (NSCLC). SCLC diagnosis and surveillance remain significant challenges. The aberrant expression of circulating microRNAs (miRNAs/miRs) is reported in many tumors and can provide insights into the pathogenesis of tumor development and progression. Here, we conducted a comprehensive assessment of circulating miRNAs in SCLC with a goal of developing a miRNA-based classifier to assist in SCLC diagnoses.

**Methods:**

We profiled deregulated circulating cell-free miRNAs in the plasma of SCLC patients. We tested selected miRNAs on a training cohort and created a classifier by integrating miRNA expression and patients’ clinical data. Finally, we applied the classifier on a validation dataset.

**Results:**

We determined that miR-375-3p can discriminate between SCLC and NSCLC patients, and between SCLC and Squamous Cell Carcinoma patients. Moreover, we found that a model comprising miR-375-3p, miR-320b, and miR-144-3p can be integrated with race and age to distinguish metastatic SCLC from a control group.

**Discussion:**

This study proposes a miRNA-based biomarker classifier for SCLC that considers clinical demographics with specific cut offs to inform SCLC diagnosis.

## 1 Introduction

In the United States, lung cancer is the leading cause of cancer-related death in both women and men (1). Lung cancers are histologically classified as Small Cell Lung Cancer (SCLC) or Non-Small Cell Lung Cancer (NSCLC). Although SCLC constitutes the minority of lung cancer cases, it represents an aggressive form of cancer characterized by a high growth fraction, early development of metastases, and extremely poor prognosis, with less than 7% 5-year survival rate (2). Low-dose computed tomography (LDCT) screening among high-risk populations remains an effective strategy for curbing mortality, with an observed 20% reduction in lung cancer-associated mortality (3). However, these results appear to be primarily applicable to NSCLC cases and the strategies for SCLC diagnoses are limited to biopsy, an invasive procedure at times impacted by tissue of poor quality or quantity (4, 5). New diagnostic methods to inform diagnosis and surveillance have the potential to impact SCLC outcomes.

Liquid biopsy has emerged as a potential approach for guiding clinical decision-making in both early detection and in guiding therapies in cancers (6, 7). Multiple components of biological fluids are being investigated as potential disease-related markers, including microRNAs (miRs, miRNAs) (8). These single-stranded non-coding RNAs drive the post-transcriptional repression of gene expression (9) and can be released to the extracellular environment either as circulating cell-free molecules or encapsulated within extracellular vesicles (EVs). These vesicles are constitutively secreted by all cell types and EVs released from cancer cells can functionally alter recipient cells by reprogramming them to become active contributors to tumor growth, metastasis, and immunosuppression (10). Circulating cell-free and EV-contained miRNAs are stable and easily detectable in bodily fluids (11). Thus, circulating miRNA signatures can potentially reveal clinically relevant information about disease pathobiology and prognosis. To date, miRNAs have been considered promising candidates as circulating biomarkers in lung cancer. The majority of circulating cell-free and EV-contained miRNAs have been examined in NSCLC subtypes with only a few similar studies in SCLC. A recent study reported a miRNA panel that discriminated SCLC from NSCLC (12), while another described the expression of miR-92b and miR-375 as prognostic factors for SCLC (13). Despite the good performance of these miRNAs as possible biomarkers, neither study integrated clinical demographics in the development of the classifier nor provided specific cutoffs that could be used to assist with diagnosis, thus, limiting their clinical applicability. In this study, we tested and validated the expression of circulating miRNAs in SCLC patients and built a miRNA-based classifier.

## 2 Results

### Identification of candidate miRNAs in circulation

**Figure 1:**
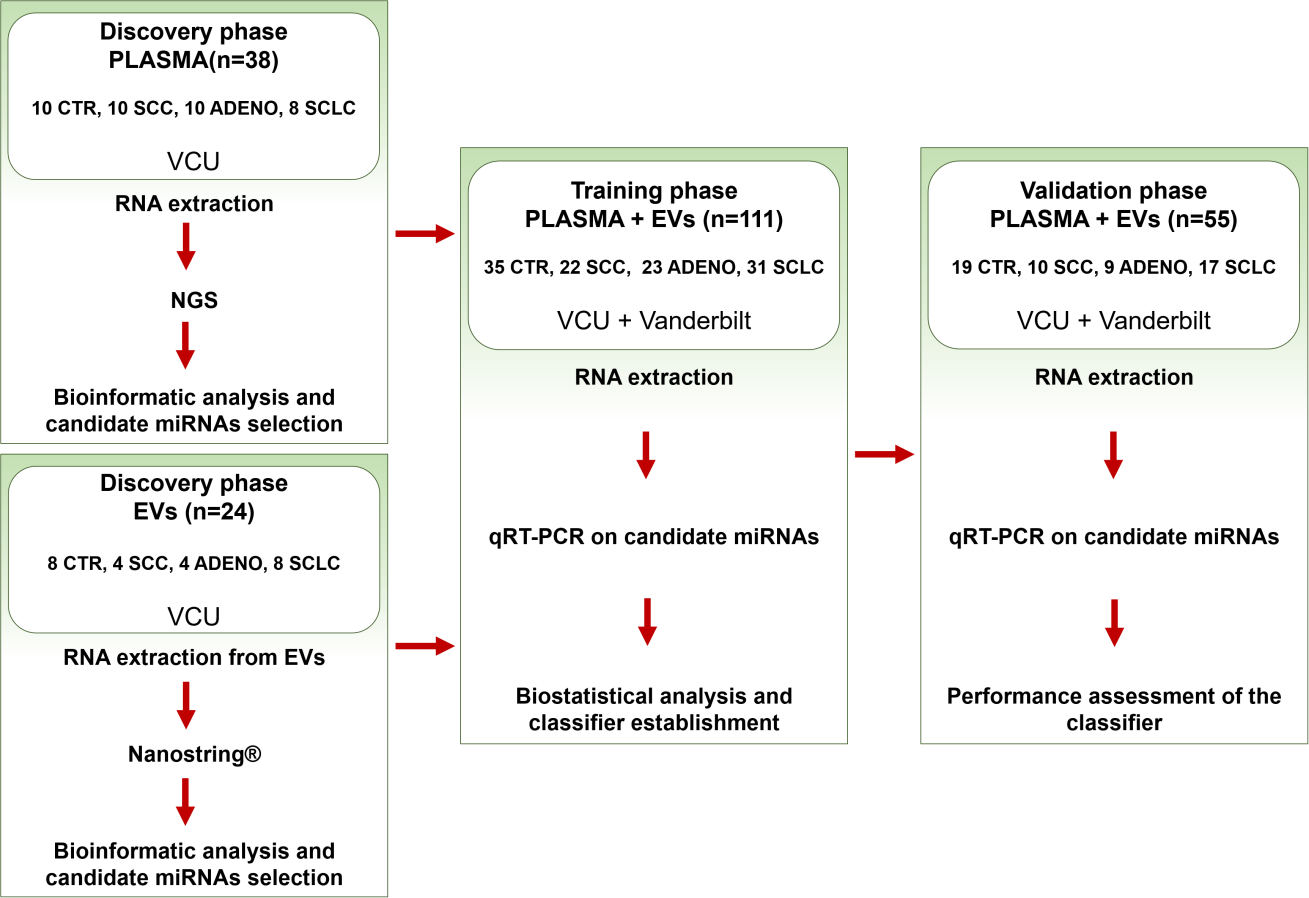
Study design for the identification of a circulating miRNA biomarker in SCLC. CTR (control group), SCC (squamous cell carcinoma), ADENO (adenocarcinoma), SCLC (small cell lung cancer). The plasma samples were obtained from VCU (Virginia Commonwealth University) and Vanderbilt medical centers.

With the intent of building a miRNA-based biomarker classifier for SCLC diagnosis (**Figure 1**, see method section), we performed RNA-seq on a discovery cohort (**Supplementary Table 1**) of 38 RNA plasma samples. We compared control (CTR), Adenocarcinoma (ADENO), Squamous Cell Carcinoma (SCC), and SCLC groups and profiled the expression of circulating cell-free miRNAs (constituted by both cell-free and EVs included miRNAs). The sequencing was focused on small RNA profiling and revealed deregulation of several miRNAs in SCLC patients compared to the other histological groups. We specifically found that plasma miR-375-3p was significantly upregulated in the SCLC group compared to the other groups (**Supplementary Table 2** and **Supplementary File 1**).

To improve the distinction between SCLC and the other histological types, a second analysis was conducted on the RNA-seq data to identify additional deregulated miRNAs. For this purpose, we chose those miRNAs with low P-value (<0.05) in at least one comparison (SCLC vs CTR, SCLC vs ADENO, SCLC vs SCC, SCLC vs NSCLC, SCLC vs OTHER) and/or with low correlation with miR-375-3p. Among these, we elected to test miR-122-5p, miR-144-3p, miR-145-5p, miR-200a-3p, miR-200b-3p, miR-205-5p, and miR-320b (**Table 1** and **Supplementary File 2**).

**Table 1:** circulating cell-free miRNAs selected from NGS RNA-seq analysis.

miR	KW raw p	KW FDR	SCLC vs ADENO raw p	CTR vs SCLC raw p	SCC vs SCLC raw p	SCLC vs OTHER raw p	SCLC vs NSCLC raw p	Correlation with miR-375-3p
**miR-375-3p**	**0.0001**	**0.0281**	**0.0076**	**0.0002**	**0.0006**	**0.0001**	**0.0004**	1
**miR-200a-3p**	**0.0136**	0.7668	0.9291	**0.0140**	**0.0142**	0.0549	0.1857	0.484
**miR-200b-3p**	**0.0290**	0.8626	0.7220	**0.0474**	**0.0359**	0.1392	0.3329	0.434
**miR-122-5p**	**0.0423**	0.9967	0.0676	0.3704	0.4598	0.4352	0.5661	0.178
**miR-144-3p**	**0.0468**	0.9967	**0.0343**	0.6730	0.4082	0.2994	0.0887	0.053
**miR-205-5p**	0.0902	0.9967	0.1972	0.3114	0.3279	0.5795	0.1857	-0.002
**miR-145-5p**	0.0923	0.9967	0.9292	**0.0268**	0.2662	0.2029	0.5757	0.375
**miR-320b**	0.1424	0.9967	0.8286	**0.0464**	0.1011	0.1169	0.2806	0.383

In bold are shown the p-values <0.05..

In parallel, we profiled EV-included miRNAs by NanoString in a second discovery cohort of 24 patients (**Supplementary Table 1**). The bioinformatic analysis identified few miRNAs upregulated in SCLC. Among these, we selected only miR-1285-5p, which was present in all the comparisons and showed a fold change higher than 1.5 in at least one comparison (**Supplementary File 3**). Moreover, considering the relevant upregulation of miR-375-3p in plasma, which contains also EVs, we decided to investigate its expression specifically in EVs.

### Testing of candidate miRNAs

We evaluated the expression of the selected miRNAs by qRT-PCR in a training cohort of 111 samples (**Table 2**), from which we extracted both circulating cell-free miRNAs in whole plasma and EV-included miRNAs.

**Table 2:** Characteristics of patients in Training and Validation cohorts.

		Training cohort (n=111)								Validation cohort (n=55)							
		CTR (n=35)		SCC (n=22)		ADENO (n=23)		SCLC (n=31)		CTR (n=19)		SCC (n=10)		ADENO (n=9)		SCLC (n=17)	
**Age ± SD**		60 ± 9		67 ± 8		65 ± 10		66 ± 9		60 ± 12		65 ± 10		64 ± 8		61 ± 13	
**Sex**	**F**	15	43%	10	45%	9	39%	10	32%	7	37%	5	50%	4	44%	6	35%
	**M**	20	57%	12	55%	14	61%	21	68%	12	63%	5	50%	5	56%	11	65%
**Race**	**B**	5	14%	8	36%	6	26%	7	23%	0	0%	3	30%	5	56%	4	24%
	**W**	30	86%	14	64%	17	74%	24	77%	19	100%	7	70%	4	44%	13	76%
**Smoking status**	**Never smoked**	5	14%	2	9%	3	13%	1	3%	4	21%	1	10%	0	0%	0	0%
	**Former smoker**	15	43%	10	45%	10	43%	13	42%	7	37%	4	40%	7	78%	5	29%
	**Current smoker**	15	43%	10	45%	10	43%	17	55%	8	42%	5	50%	2	22%	12	71%
**Pk-Yr Hx ± SD**		43 ± 21		59 ± 32		45 ± 25		49 ± 26		54 ± 25		57 ± 38		41 ± 32		48 ± 29	
**TNM**	**I-II**			10	45%	8	35%	9	29%			4	40%	4	44%	4	24%
	**III**			7	32%	9	39%	8	26%			3	30%	2	22%	6	35%
	**IV**			5	23%	6	26%	14	45%			3	30%	3	33%	7	41%

CTR, control; SCC, squamous cell carcinoma; ADENO, adenocarcinoma; SCLC, small cell lung cancer; F, female; M, male; W, white; B, black. Pk-Yr Hx, pack-year history; TNM, tumor-node-metastasis stage.

Due to the high variability existing between human samples, we used three different endogenous miRNAs to normalize the expression of the circulating cell-free miRNAs, resulting in three features in the selection pool for each of these miRNAs. An exogenous spike-in was used as normalizer for EV-included miRNAs. Circulating cell-free miR-200b-3p was removed from the analysis because of its low expression (CT values >35 in more than 10% of samples).

**Figure 2:**
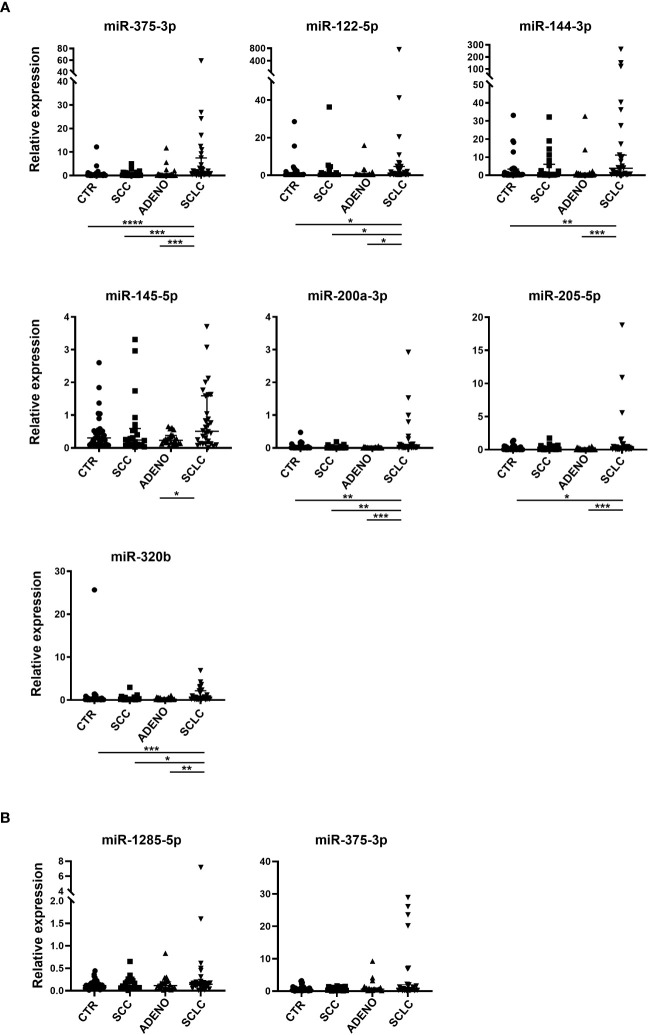
Relative expression of selected circulating cell-free miRNAs normalized with miR-24-3p **(A)** and miRNAs included in EV **(B)** in the training cohort. Mann Whitney P-values: *=p<0.05; **=p<0.01; ***=p<0.001; ****=p<0.0001.

Based on qRT-PCR data, we identified 7 circulating cell-free miRNAs significantly upregulated in the SCLC group in at least one comparison (**Figure 2A**). miR-375-3p, was significantly upregulated in SCLC group compared with the other groups (SCLC vs CTR: p<0.0001; SCLC vs SCC and SCLC vs ADENO: p<0.001); miR-122-5p was significantly upregulated in SCLC vs all the other groups (p<0.05); miR-144-3p was upregulated compared to CTR (p<0.01) and ADENO (p<0.001); miR-145-5p was upregulated in SCLC vs ADENO group (p<0.05); miR-200a-3p was upregulated in SCLC vs all the other groups (SCLC vs CTR and SCLC vs SCC: p<0.01; SCLC vs ADENO: p<0.001), miR-205-5p was upregulated in SCLC vs CTR (p<0.05) and SCLC vs ADENO (p<0.001); miR-320b was upregulated in SCLC vs all the other groups (SCLC vs CTR: p<0.001; SCLC vs SCC: p<0.05; SCLC vs ADENO: p<0.01).

By contrast, none of the tested EV-miRNAs showed significant deregulation in SCLC group (**Figure 2B**).

### Establishment and validation of the classifier

With the intent of building a classifier capable of distinguishing SCLC from the other histological subtypes (SCC, ADENO and CTR), we employed LASSO regression for selecting the variables (miRNAs expression and clinical data). Acknowledging the high expression of circulating cell-free miR-375-3p, and its significant upregulation in SCLC, we forced its expression into the model. We also forced the batch effect in the classifier, to consider the variability between the sample processing placed in the two different medical centers from which we obtained the samples. We first tested the association between clinical variables (age, sex, race, smoking history, histology, stage) and miR-375-3p expression to ensure that the miRNA expression was independent from the clinical data. We found that the only association of miR-375-3p expression was related to histology and stage.

We estimated the model on the complete training dataset (see methods), and then we evaluated its diagnostic performance on the qRT-PCR results for both the circulating cell-free and EV-included miRNA of the validation data set (**Supplementary Figure 1**). For each comparison, we estimated the probability of being SCLC by using area under the receiver operating characteristics (ROC) curve (AUC), setting a specific cutoff, calculating specificity, sensitivity, positive predictive value (PPV), and negative predictive value (NPV) at the optimal cutpoint derived from the training data (**Table 3**).

**Table 3:** Variables selected for each comparison and performance assessment of the classifier.

Comparison	Retained variables	Coefficient	Cutpoint	AUC	Sensitivity	Specificity	PPV	NPV
SCLC vs CTR	Batch	0.634	0.4166	0.574	0.3125	0.8824	0.7143	0.576
	Age	0.3153						
	PLASMA.miR.122.5p.vs.93.5p	-0.01554						
	PLASMA.miR.320b.vs.93.5p	-0.003632						
	PLASMA.miR.375.3p.vs.126.3p	2.573						
SCLC vs ADENO	Batch	1.091	0.5771	0.75	0.375	1	1	0.411
	PLASMA.miR.375.3p.vs.24.3p	0.5744						
SCLC vs SCC	Batch	1.013	0.5494	0.833	0.375	1	1	0.375
	PLASMA.miR.375.3p.vs.126.3p	1.507						
SCLC vs NSCLC	Batch	0.9479	0.4012	0.793	0.375	1	1	0.565
	PLASMA.miR.375.3p.vs.24.3p	0.3593						
SCLC vs OTHER	Batch	0.9766	0.4423	0.658	0.3125	1	1	0.731
	PLASMA.miR.144.3p.vs.24.3p	0.003482						
	PLASMA.miR.375.3p.vs.126.3p	1.206						
SCLC stage I+II vs CTR	Batch	2.17	0.2394	0.515	0.5	0.5294	0.2	0.818
	PLASMA.miR.375.3p.vs.126.3p	-4.265						
SCLC stage I+II+III vs CTR	Batch	0.5922	0.3019	0.444	0.4444	0.4118	0.2857	0.583
	PLASMA.miR.375.3p.vs.126.3p	1.239						
SCLC stage IV vs CTR	Batch	-0.1454	0.3414	0.882	0.2857	0.9412	0.6667	0.761
	Age	0.4699						
	Race	-0.6286						
	PLASMA.miR.144.3p.vs.24.3p	0.01695						
	PLASMA.miR.320b.vs.126.3p	-3.508						
	PLASMA.miR.375.3p.vs.126.3p	6.354						

AUC, area under the ROC curve; PPV, positive predicted value; NPV, negative predicted value.

**Figure 3:**
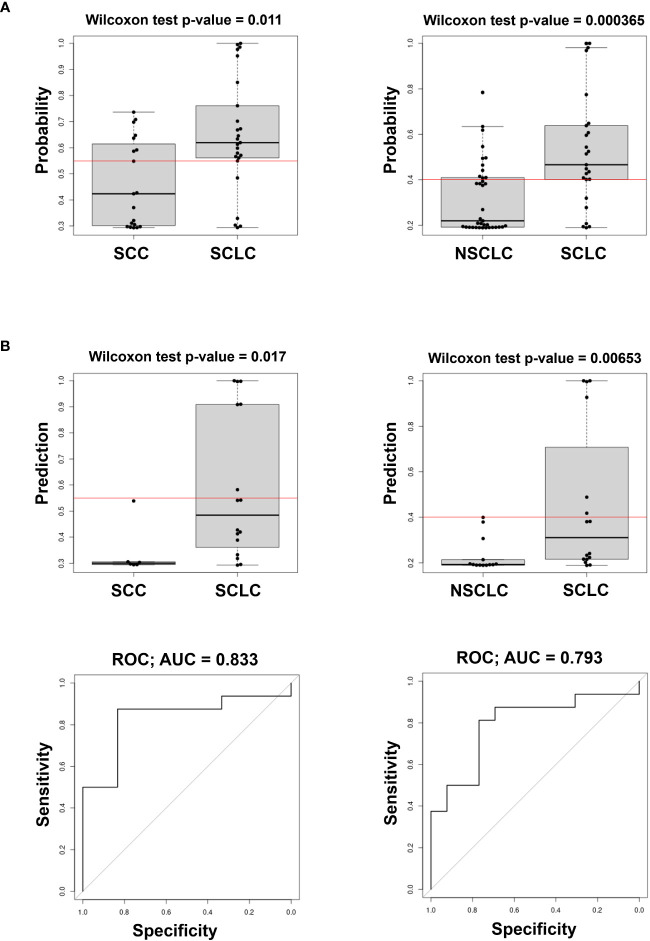
Predicted probability of SCLC in comparison of SCLC vs SCC (left) and SCLC vs NSCLC (ADENO + SCC) (right) in Training **(A)** and Validation cohorts (**B** top) and ROC curves (**B** bottom) of the model in the Validation cohort. The Red line in box plots indicates the optimal cut-point derived from the training data.

We also evaluated the predicted probability of SCLC association with the group in the validation dataset. Our data show that the variables selected comprise circulating cell-free miRNAs, whereas none of the EV-included miRNAs were retained in the model. As shown in **Figure 3**, the model built on the training cohort provided a significant classification between SCLC patients and NSCLC (SCC + ADENO) and between SCLC and SCC patients in the validation dataset, highlighting its diagnostic relevance. In the SCLC vs SCC classification, miR-375 turned out to be an effective predictor, with AUC= 0.833 and with sensitivity and specificity 0.375 and 1, respectively. The applied cutoff was 0.5494 which discriminated, in the validation dataset, 6 out of 16 test-positive SCLC patients and 0 out of 6 test-positive SCC patients (**Supplementary Table 3**), ruling out any SCC from having a positive test. As well, in SCLC vs NSCLC classification, miR-375-3p was effective predictor of SCLC, with AUC= 0.793 with sensitivity and specificity 0.375 and 1, respectively. The set cutoff was 0.4012 which correctly discriminated 6 out of 16 test-positive SCLC patients, and 0 out of 13 test-positive NSCLC patients. The results of the remaining comparisons show a significant classification in the training dataset, however, the performance of the classifier in validation data was not significant with a low AUC (**Supplementary Figure 2**, **Table 3**). Taken together, these results demonstrate that levels of circulating cell-free miR-375-3p can provide a significant classification between SCLC and NSCLC patients.

To evaluate the usefulness of our classifier in informing stage, we compared early stage (stages I+II), non-metastatic (stages I+II+III), and metastatic (stage IV) SCLC with the CTR (**Supplementary Figure 3**). We found that miR-375-3p, miR-320b, and miR-144-3p combined with race and age could discriminate between stage IV SCLC patients and the CTR group (AUC=0.882, sensitivity=0.2857, and specificity =0.9412).

## 3 Discussion

The prompt diagnose of SCLC is essential for effective treatment as the majority of patients present with advanced disease (14). Further, given the high rates of recurrence of the disease, classifiers that could be applied to surveillance strategies would be invaluable. MiRNAs are small molecules that are unique for their stability and detection in body fluids, making them potentially valuable biomarker candidates (15). Sozzi et al., reported that a combination of both plasma miRNA signature and LDCT resulted in the reduction of LDCT false-positive rate (16), highlighting the utility of miRNA in informing lung cancer diagnoses. Despite investigation describing miRNAs deregulation in lung cancers (17), few studies have identified a panel of miRNAs distinguishing SCLC from NSCLC (12, 18). NSCLC represents the majority of lung cancer cases while SCLC remains a less common subtype of tumor that is often not considered in biomarker studies. SCLC is an exceptionally aggressive tumor type that rapidly becomes metastatic and chemo resistant thus carrying poor prognosis (2). Moreover, compared to NSCLC that is often characterized by mutations of targetable oncogenes, SCLC frequently exhibits gene alterations in the tumor suppressors P53 and RB (89% and 64% respectively), which are near-universally inactivated (14, 19), hindering the development of targeted therapies. The neuroendocrine features and the high heterogeneity of the tumor, render SCLC a completely different cancer compared to NSCLC in terms of pathology, progression, and possible therapeutic treatments (20, 21). Importantly, the rapid rate of proliferation is reflected in the late presentation of the disease, when the tumor is unresectable, and the biopsy may be obtained from a sample of poor quantity or quality (4, 22). For this reason, a thorough understanding of specific circulating markers capable of detecting SCLC in a cost-effective and non-invasive way would be invaluable.

Several miRNAs regulating proliferation, metastasis and chemoresistance have been detected in SCLC and are suggested as candidate biomarkers for monitoring response to chemotherapy and predicting survival outcomes (23, 24). However, although the expression of miRNAs in circulation has been reported for cancer diagnosis, they have yet to reach effective clinical utility. Our work represents a step towards developing a miRNA-based classifier to distinguish SCLC from SCC and NSCLC.

In this study, we used a total of 166 plasma samples from lung cancer patients to identify miRNA deregulation across histological subtypes. To profile circulating free miRNAs from whole plasma, we used NGS, a sophisticated and highly informative technique that provides the exact miRNA sequence and can potentially inform about the presence of miRNA isoforms (25, 26). To profile miRNAs included in EVs, we elected to use NanoString, given our previous experience in analyzing miRNAs included in EVs with this technology (27–29). Although less informative compared to NGS, this technique allows for minimal sample processing and easier data analysis. In fact, NanoString technology does not require any preamplification process, accurately counts the number of molecules in a specific sample and has good performance for low-expressing miRNAs (30). Finally, we used qRT-PCR to test selected miRNAs and build the classifier for SCLC diagnosis. qRT-PCR is extensively used for assessing the miRNAs expression; moreover, it is a reliable and cost-effective technique that can be easily used for routine diagnostics.

qRT-PCR on samples derived from EVs did not show significantly deregulated miRNAs in SCLC patients (**Figure 2**). This result was not entirely unexpected and may depend on the low fold change of the selected miRNAs which did not allow for accurate discrimination between groups. Numerous studies have suggested EV-based miRNAs as biomarkers in cancer (31). However, EV isolation requires several steps and a specific spike-in is essential for normalization issues (32), limiting their clinical relevance and reproducibility.

We observed a significant upregulation of circulating cell-free miR-375-3p in SCLC compared to the other groups (**Figure 2**). Using LASSO regression for selecting variables, we evaluated and validated a model to discriminate SCLC from NSCLC and, more precisely, between SCLC and SCC (**Figure 3**). The importance of this discrimination lies in the radiographic similarity between SCLC and SCC, both of which arise centrally in the lung, hindering their distinction by imaging. Furthermore, histologically, SCC tumors may occasionally show characteristics in common with SCLC, such as small cells with hyperchromatic nuclei and scant cytoplasm, leading to misdiagnose (5). Thus, a non-invasive approach for additional discrimination could be of value.

An upregulation of miR-375 in SCLC was previously reported by Lu et al. in a large cohort of patients (12). We obtained similar results in a relatively small cohort of patients but included clinical demographic variables to build a more comprehensive and accurate classifier for SCLC. Furthermore, we provide specific cut points for miR-375 expression in each comparison which represents the first step towards potential clinical application for informing SCLC diagnoses.

The expression of miR-375 in SCLC is highly variable and, occasionally, overlaps with those of other histological types. The variability in miR-375 expression in SCLC may be attributable to the presence of different SCLC subtypes which may harbor variable expression of this miRNA. Recently, gene expression profiling of SCLC patients, cell lines, and mouse models revealed four major SCLC subtypes (33), distinguished by four major transcription factors: ASCL1, NEUROD1, YAP1, and POU2F3, which differ for neuroendocrine grades. ASCL1 is known to induce miR-375 transcription in lung cancer and its expression is associated with elevated neuroendocrine characteristics in lung cancer (34, 35). Interestingly, miR-375 targets YAP1 (36), suggesting a role of this miRNA as a mediator of neuroendocrine differentiation and tumorigenesis in lung carcinoid cells (37). The expression of miR-375 in our classifier did not discriminate between SCLC and adenocarcinoma patients (**Supplementary Figure 2**). High expression of miR-375 in lung adenocarcinoma has been recently reported by Kumar et al. (38) consistent with our findings. As such, Augustyn et al. showed that ASCL1 is expressed in 8% of lung adenocarcinomas, which plays a tumor-promoting role (39) and could partially explain the upregulation of miR-375 in the plasma of some NSCLC patients (34). Conversely, a recent paper showed that miR-375 is often downregulated in NSCLC tissues compared to adjacent tissues (40), and that the overexpression of this miRNA induces cisplatin sensitivity in lung adenocarcinoma cell lines (41).

MiR-375 is downregulated in several cancers, where it acts as tumor-suppressor by targeting oncogenes like PDK1, and IGFR1 and by suppressing the PI3K/Akt pathway (42, 43). However, its upregulation has been reported in other tumors, such as breast cancer, where circulating miR-375 can be internalized by tumor-associated macrophages altering their phenotype to create a pro-tumoral environment (44). The variable function of miR-375 in cancer reflects a tumor specific role of this miRNA that has yet to be fully elucidated.

A recent study demonstrated that exosomal miR-375 induces brain metastasis in SCLC by targeting CLAUDIN-1 (45). Consistent with these findings, our classifier could distinguish metastatic SCLC from control patients. Although the small number of samples requires further validation, this result suggests that the selected variables can potentially provide insight into metastatic disease and tumor burden.

To the best of our knowledge, we have validated for the first time a miRNA-based biomarker classifier capable of distinguishing between SCLC and SCC. In addition, we established a classifier specific to stage in SCLC by combining circulating cell-free miRs and clinically relevant patient information. Building a classifier should require consideration of clinical variables which are not often included in other studies. We believe that including the patients’ demographics is fundamental for clarifying the risk factors for SCLC. It is known that the expression of miRNAs can be influenced by demographic factors such as sex, race, age, and smoking history, and discrepancy in miRNA profiling in different sex and race were previously observed (46–50). We found no association between the expression of miR-375 and the patients’ demographics, demonstrating that miR-375 may be related to SCLC histology. It is important to mention that incorporating patients’ clinical information helps to establish a correct classification including minorities that have different lung cancer incidence and mortality (51, 52).

We recognize that there are limitations to our study as presented. First, given the relatively small sample size, our findings result require additional external validation as well as assessment for actual clinical utility. The optimal cutpoint for each comparison was determined based on the minimum p-value. It is important to specify that the cutpoints determined from a higher number of samples would be more precise. Although the sensitivity, specificity, PPV and NPV for the validation were computed using the cutpoints derived from the training data, considering the limited number of patients in our training cohort the cutpoints are certainly parameters that would need to be refined prior to consideration of the model for clinical use. Moreover, our classifier for distinguishing between SCLC and SCC/NSCLC includes only miR-375. To increase the accuracy of the classification, additional clinical and genomic features should be considered. One possibility would be to investigate the expression of proteins, metabolites, or lipids. A particularly intriguing approach would be to investigate for modified miRNAs, such as isomiRNAs or edited miRNAs that could then be included in the analysis. Additionally, a more comprehensive study should take into consideration different SCLC subtypes and their correlation with miR-375 expression. Such information would increase the diagnostic relevance of biomarkers suitable for detecting specific SCLC subtypes that can be specifically targeted (33, 53–55).

Nevertheless, our findings represent a step towards the clinical utility of cell-free miRNA circulation as a reliable, cost-effective, and non-invasive biomarker that may have potential as a complement to histology for SCLC diagnosis.

## 4 Materials and methods

### Ethics statement

A total of 166 patients were selected for this study. The patients were recruited from two medical centers (Virginia Commonwealth University, Richmond, VA, USA, and Vanderbilt University, Nashville, TN, USA) in accordance with the institutional review board (IRB) for equivalent guidelines for each institution. Informed consent was obtained from all subjects and/or their legal guardian(s).

### General design

Based on previous approaches (56, 57), we conducted a three-phase study (**Figure 1**): Discovery (phase 1), Training (phase 2) and Validation (phase 3). The Discovery phase aimed to identify cell-free and EV-included miRNAs present in circulation that were deregulated in the SCLC group compared to the other histological types: control (CTR) which includes normal and granuloma histology, squamous cell carcinoma (SCC), and adenocarcinoma (ADENO). We used plasma samples from a first discovery cohort collected at Virginia Commonwealth University (VCU) (n=38, **Supplementary Table 1**) to extract circulating cell-free miRNAs that were then analyzed by Illumina Next Generation Sequencing (NGS). These samples include both circulating free miRNA as well as EV-included miRNA. In parallel, we isolated EVs from a second discovery cohort (n=24, **Supplementary Table 1**) and extracted EV-included miRNAs, which were then analyzed through NanoString.

Accurate isolation of EVs was confirmed by Nanoparticle Tracking Analysis (NTA) and cryo-electron microscopy (cryo-EM), as suggested by the International Society of Extracellular Vesicles (58) (**Supplementary Figure 4**).

In the Training phase, we extracted both circulating cell-free and EV-included miRNAs from a larger cohort (training cohort, **Table 2**, left), which included part of the samples from the discovery cohort. From these samples, we tested, by qRT-PCR, candidate miRNAs in both plasma and EVs.

A biomarker classifier for detecting SCLC was established by using the expression of circulating cell-free miRNAs and EV-contained miRNAs from paired samples, as well as six clinical variables (histology, stage, sex, race, smoking history, and age), in the selection pool for the LASSO model in the training cohort.

In the Validation phase, we first evaluated the expression of circulating cell-free and EV-included miRNAs by qRT-PCR on an independent validation cohort (**Table 2**, right), which included part of the samples from the discovery cohort. Finally, we applied of the model generated on the training data set to the validation data set.

### Patient selection and plasma preparation

The plasma samples were divided into two clinically homogeneous groups, identified as training (n=111) and validation (n=55) (59, 60). To ensure comparable training and validation sets, splits of 111/55 were performed randomly 10,000 times, and group (training/validation) was tested for association with each of six clinical variables (histology, stage, sex, race, smoking history, and age) at each split. The split with the highest minimum p-value (p>0.9) across the six variables was retained for the training and validation analyses.

Plasma samples were spun at 2,000 X g for 20 minutes and subsequently at 10,000 X g for 20 minutes to remove cellular debris. 200 µl of supernatant were used for whole plasma miRNAs extraction and 500 µl were used for EVs isolation.

### EV isolation

EVs were isolated from plasma using the Total Exosome Isolation Kit (INVITROGEN #4484450) following the manufacturer’s protocol. EVs were thoroughly resuspended in 200 µl of PBS with 1/10 reserved for EVs characterization.

### Nanoparticle tracking analysis

Nanoparticle tracking analysis was performed using the NanoSight NS300 system (Malvern, Great Malvern, UK) as previously described (61).

### Cryo-EM

EV characterization by cryogenic transmission electron microscopy (cryo-EM) was performed at the Advanced Materials and Liquid Crystal Institute (AMLCI), Kent State University, OH, USA. A FEI Vitrobot (Mark IV) plunge freezer was used to prepare vitrified cryo-TEM specimens from the solution sample (62). Cryo-TEM observation was performed on a FEI Tecnai F20 transmission electron microscope. The basic experimental setup and procedure can be found in (63).

### RNA extraction and qRT-PCR from patients’ samples

Circulating cell-free RNA was extracted by using the miRNeasy micro kit (QIAGEN #217084) according to the manufacturer’s protocol. RNA from EVs was extracted using TRIzol reagent (Invitrogen #15596018) and supplied with 35 picograms of non-human miRNA spike-ins (ath-miR-159a; cel-miR-248; osa-miR-414), for normalization purposes. RNA purification was performed with RNA Clean-Up and Concentration Kit (NORGEN #43200). 3 µl of RNA from plasma and EV samples were retrotranscribed using the TaqMan® Advanced miRNA cDNA Synthesis Kit (#A28007) and qRT-PCR was performed using TaqMan® reagents (TaqMan™ Advanced miRNA assay #4444964; TaqMan™ Fast Advanced Master Mix # 4444557, Thermo Fisher). All the probes used were obtained from TaqMan® (#A25576). MiRNAs 24-3p, 93-5p and 126-3p were used as endogenous controls for normalizing whole plasma PCR data, as suggested from the manufacturer; ath-miRNA-159a was used as a unique normalizer for EV PCR data. All the assays were carried out in three technical replicates. Samples and technical replicates in which the Ct value was >35 were excluded from the statistical analysis. The 2^-dct values were used as relative expression of the biostatistical analysis.

### NGS and feature selection analysis

NGS was performed at the Institute for Systems Biology, Seattle, Washington as previously reported (64). One of the control samples was considered an outlier and removed from the NGS analysis. Raw fastq files were initially pre-processed by trimming Illumina adapter sequences via Cutadapt tool (65). and then qualitatively filtered by Condetri tool (66) (parameters: -pb=fq -lq=20 -hq=30 -minlen=15 -sc=33). For each sample, all post-processed reads were mapped into the human genome (GRCh37) by using miARma-Seq tool (67), which includes Bowtie tool (v1) (68) for aligning reads and featureCounts tool (69) to annotate them into microRNA space [miRBase v20 (70)]. A minimum expression filtering is applied prior to normalization, retaining all those miRNA molecules with at least 10 reads in 50% of samples. Filtered miRNA were normalized by applying the TMM method (trimmed mean of M-values). Differentially expression analysis was carried out by using EdgeR package (71). For each comparison, we took into consideration all those miRNAs with a P-value<.05 and |Linear Fold-Change|>1.5 (**Supplementary File 1**). For a second more restricted analysis in the discovery data, features were filtered by percent present >90%, and at least 15 read counts in a least 50% of the samples in at least one group. Count data were analyzed across groups with the omnibus Kruskal-Wallis test, followed by pairwise by group analysis with the Wilcoxon rank sums tests. Spearman correlation was used to determine miRNA/miRNA associations. Additional miRNAs were selected for inclusion in the classification stage by association with group and low correlation with miR-375 (**Supplementary File 2**).

### NanoString nCounter assay and feature selection analysis

Total exosomal RNA from a cohort of 24 samples (8 CTR, 4 SCC, 4 ADENO, 8 SCLC) were profiled through NanoString nCounter Human v3 miRNA Expression Assay as previously described (29).

Raw data (.rcc files) produced via NanoString nCounter Human v3 miRNA Expression Assay were analyzed with nSolver™ (provided by NanoString Technologies). Negatives controls were considered to perform background noise subtraction, while positive controls were considered to perform technical normalization, adjusting lane-by-lane variability due to differences in hybridization, purification, or binding. Finally, data were biologically normalized by calculating the geometric mean of the top 100 miRNAs in all samples, as recommended by NanoString company. Differential expression analysis was carried out by using Limma R package (72) from the Bioconductor R project. For each comparison, we took into consideration all those miRNAs with a P-value<.05, |Linear Fold-Change|>1.25 and an average expression of >30 count in at least one condition (**Supplementary File 3**).

### Statistical analysis and predictive modeling

Training and validation analyses were performed in R (73). For qRT-PCR data, hierarchical clustering using the Ward method (74) on correlations was performed using differentially expressed miRNAs. For the training data, LASSO (glmnet package) was used to select variables and produce the final model. Data were produced in two batches, and therefore a batch variable was forced into the LASSO models. Additionally, the initial strongest association in the discovery data was miR-375-3p, and, therefore, this feature was also forced into the LASSO models. The variables considered were the log10(2^-dct) of EV- included miR-1285-5p, miR-375-3p, and circulating cell-free miR-122-5p, miR-144-3p, miR-145-5p, miR-200a-3p, miR-205-5p, miR-320b, miR-375-3p vs the normalizers miR-24-3p, miR-93-5p and miR-126-3p, as well as clinical data (race, sex, age, and smoking history).

The samples with missing features were not considered for the predictive modeling and the analysis was performed on a total of 94 (Training cohort) and 46 (Validation cohort) paired EVs and PLASMA samples.

The predicted probability of being SCLC from the model was used as a score for each sample. The optimal cutpoint by minimum p-value (OptimalCutpoints package) (75), was determined for each comparison to classify samples. The training set derived model was then applied to the validation set. The probability of SCLC was tested (Wilcoxon rank sums) for association with group (control vs. SCLC, for example), AUC calculated, and using the cutpoint determined from the training data, sensitivity, specificity, PPV, and NPV calculated to evaluate the utility of the model for classification in the validation set.

For the real-time PCR data, normal distribution was first assessed and non-parametric Mann Whitney test was performed using GraphPad Prism version 9.5.1 for Windows, GraphPad Software, San Diego, California USA, www.graphpad.com.

## Data availability statement

The raw data supporting the conclusions of this article are available on GEO (Project ID: GSE240759).

## Ethics statement

The studies involving humans were approved by Virginia Commonwealth University IRB HM2471 and Vanderbilt University IRB #000616 and IRB #030763. The studies were conducted in accordance with the local legislation and institutional requirements. The participants provided their written informed consent to participate in this study.

## Author contributions

MS: Data curation, Methodology, Visualization, Writing- original draft, Investigation. GR: Supervision, Writing- review & editing, Investigation, Methodology. JM: Data curation, Formal analysis, Software, Methodology, Writing- original draft. GN: Data curation, Software, Formal analysis, Writing- original draft. RD: Formal analysis, Software, Writing- review & editing. RT: Resources, Writing- review & editing. FC: Resources, Writing- review & editing. PL: Writing- review & editing. DM: Writing- review & editing. SA: Resources, Writing- review & editing. SD: Resources, Writing- review & editing. KW: Formal analysis, Writing- review & editing. LL: Writing- review & editing. MA: Funding acquisition, Supervision, Writing- review & editing. PN: Conceptualization, Funding acquisition, Supervision, Writing- review & editing.
