# Supplementary figures and images for: A plasma miRNA-based classifier for small cell lung cancer diagnosis
Source: Front Oncol. 2023 Oct 5;13:1255527. doi: 10.3389/fonc.2023.1255527 (PMC10585112; doi:10.3389/fonc.2023.1255527)

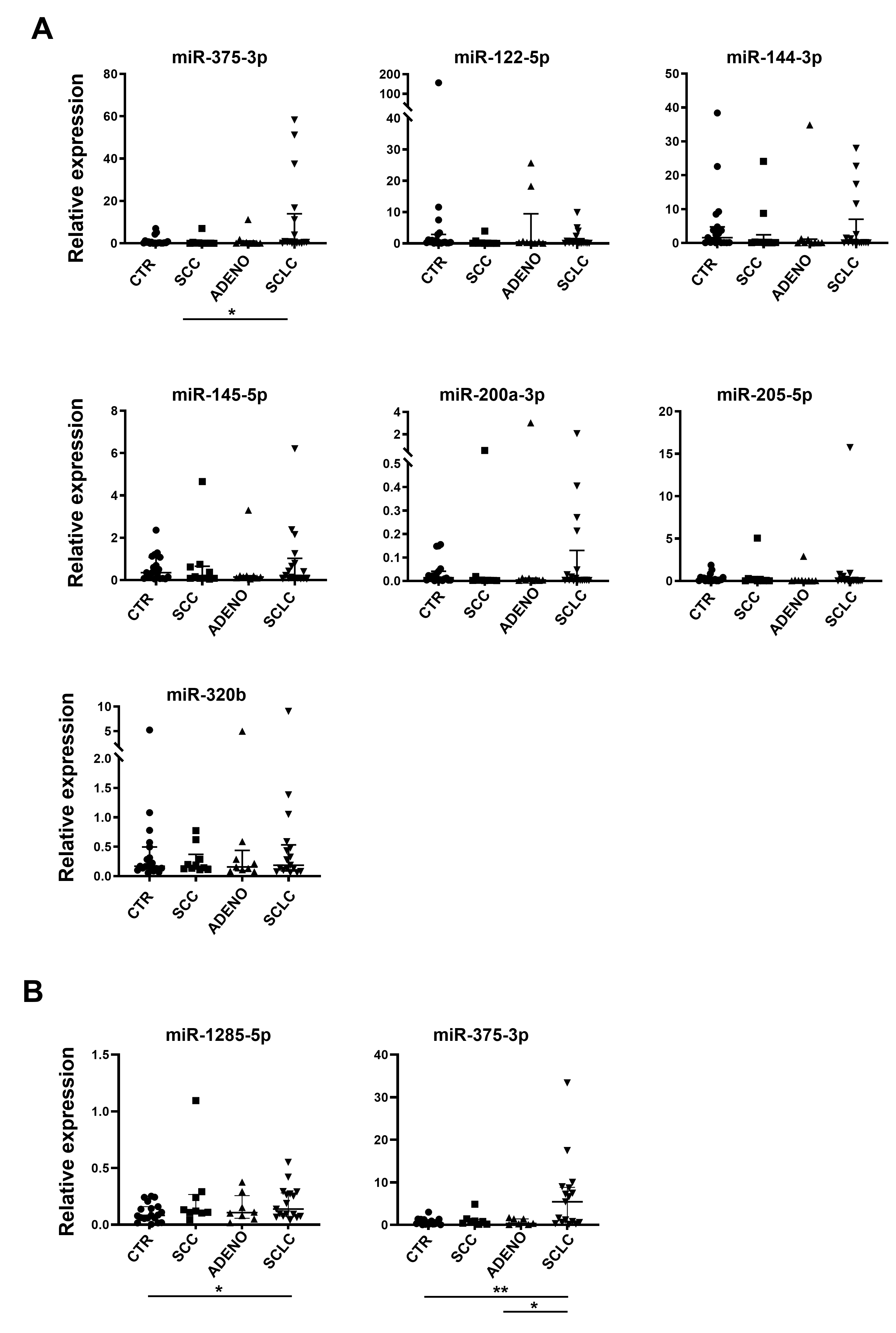

Supplement: Supplementary file 1 [file Image_1.tif]

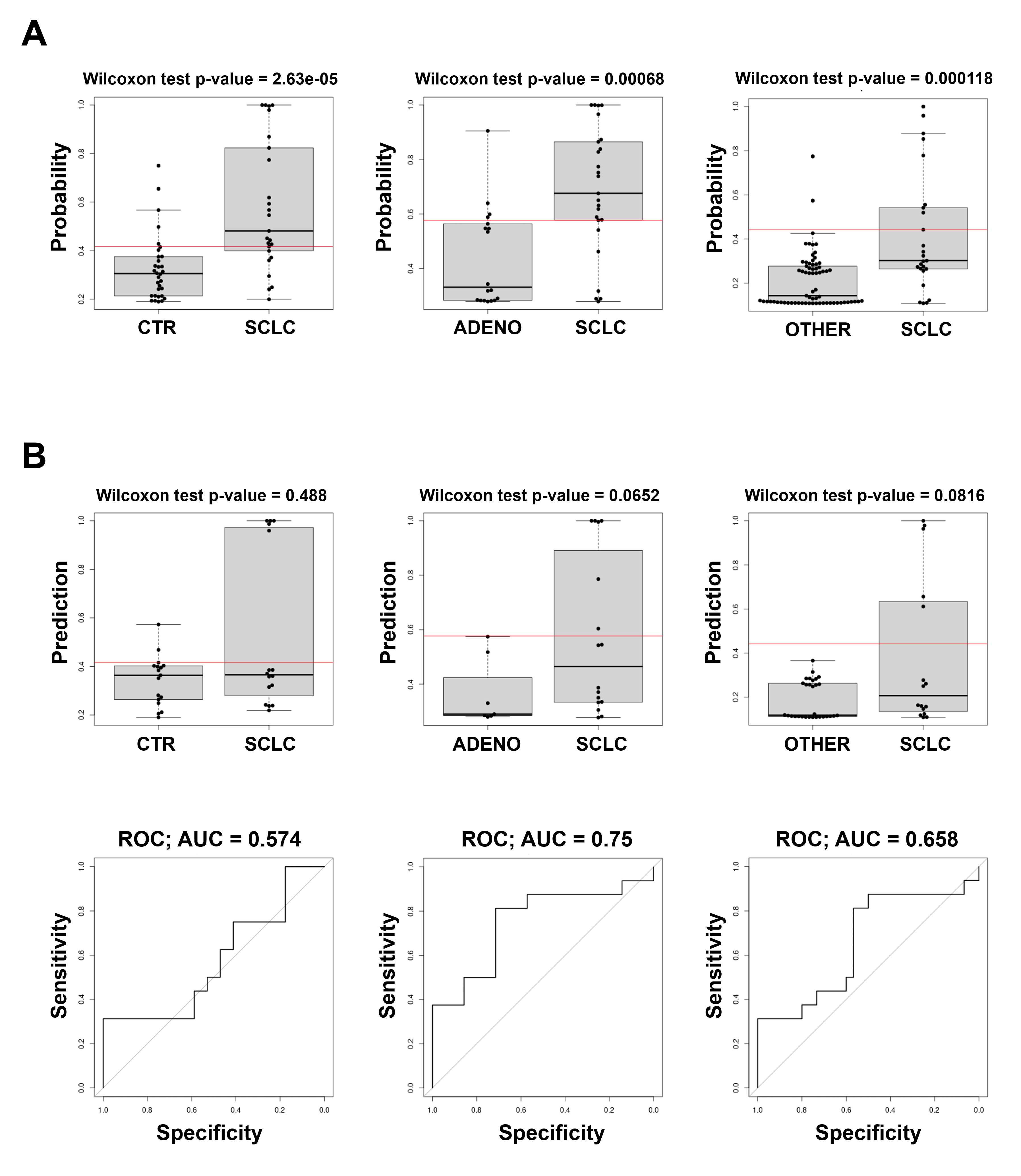

Supplement: Supplementary file 2 [file Image_2.tif]

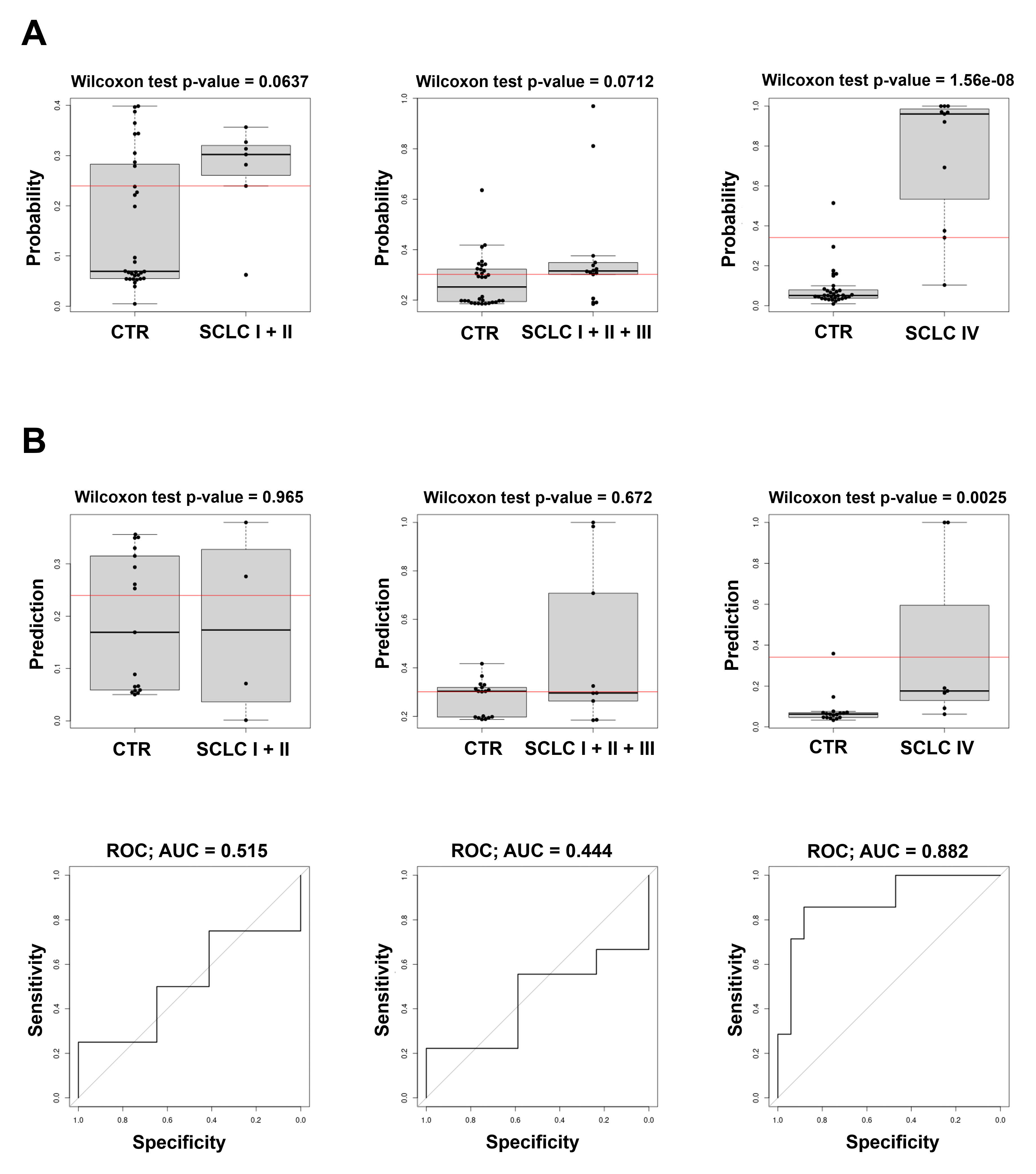

Supplement: Supplementary file 3 [file Image_3.tif]

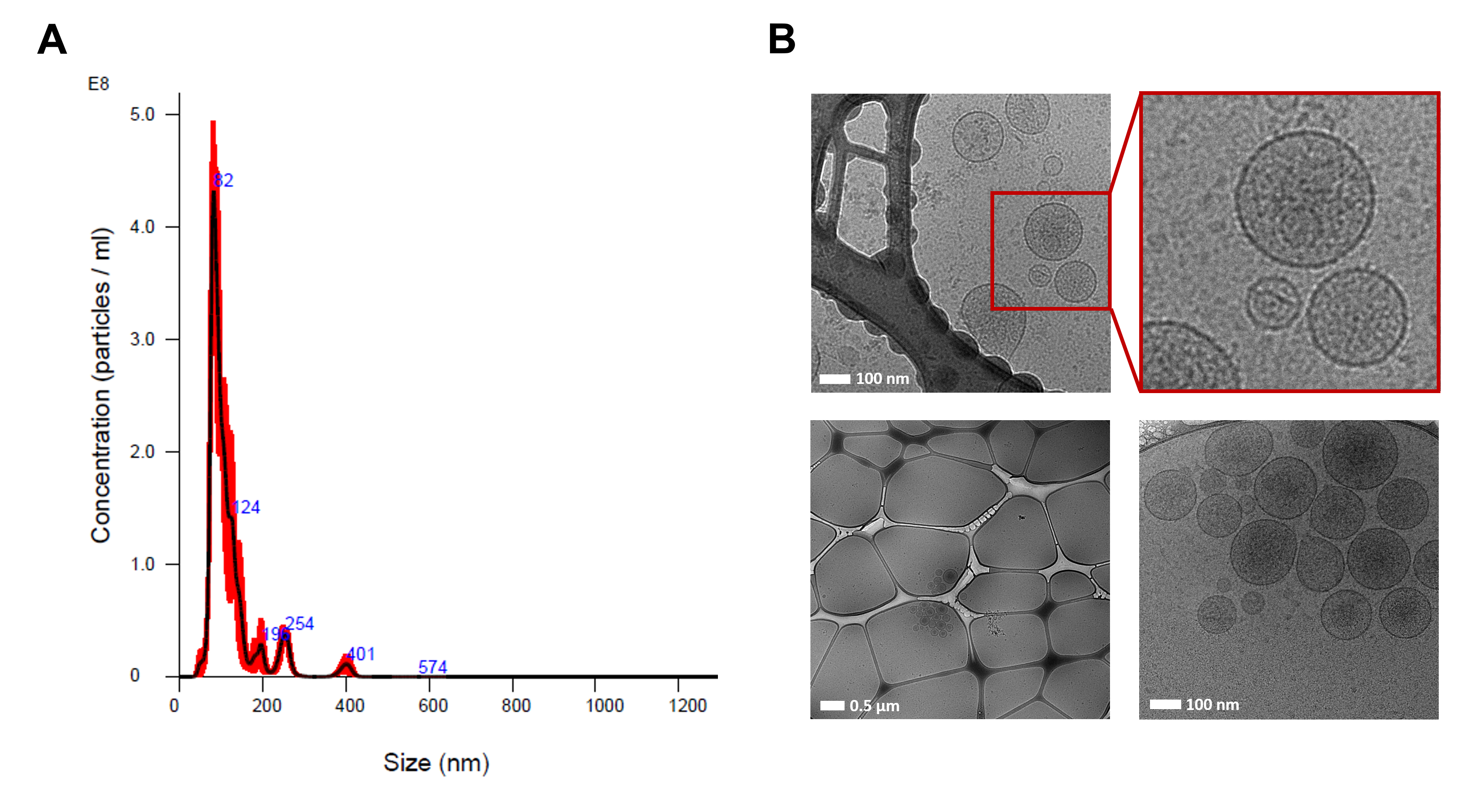

Supplement: Supplementary file 4 [file Image_4.tif]
