# Supplementary material for: A plasma miRNA-based classifier for small cell lung cancer diagnosis
Source: Front Oncol. 2023 Oct 5;13:1255527. doi: 10.3389/fonc.2023.1255527 (PMC10585112; doi:10.3389/fonc.2023.1255527)
Supplement: Supplementary file 8 [file DataSheet_4.docx]

Supplementary Material

# Supplementary Figures

**Supplementary Figure 1.** qRT-PCR in the validation cohort: Relative expression of selected circulating cell-free miRNAs normalized with miR-24-3p (A) and miRNA included in EV (B). Mann Whitney P-values: *=p<0.05, **=p<0.01.

**Supplementary Figure 2.** Predicted probability of SCLC in comparison SCLC vs CTR (left), SCLC vs ADENO (center), and SCLC vs OTHER (CTR+SCC+ADENO) (right) in training (A) and validation (B top) cohorts and ROC curves (B bottom) of the model in validation dataset. The Red line in box plots indicates the optimal cut-point derived from the training data.

**Supplementary Figure 3.** Predicted probability of SCLC in comparison SCLC stage I+II vs CTR (left), SCLC stage I+II+III vs CTR (center), and SCLC stage IV vs CTR (right) in training (A) and validation (B top) datasets and ROC curves (B bottom) of the model in validation dataset. The Red line in box plots indicates the optimal cut-point derived from the training data.

**Supplementary Figure 4.** Representative figure of extracellular vesicle’s isolation. Nanoparticle tracking analysis (A) and cryo-EM images (B).

# Supplementary Tables

**Supplementary Table 1.** Characteristics of the patients in the plasma and EV discovery cohorts. CTR= control; SCC= squamous cell carcinoma; ADENO= adenocarcinoma; SCLC= small cell lung cancer; W=white; B=black. Pk-Yr Hx= pack-year history; TNM= tumor-node-metastasis stage

|  |  | **Discovery cohort PLASMA (n=38)** | | | | | | | | | | |  |  | **Discovery cohort EVs (n=24)** | | | | | | | | | | |
| --- | --- | --- | --- | --- | --- | --- | --- | --- | --- | --- | --- | --- | --- | --- | --- | --- | --- | --- | --- | --- | --- | --- | --- | --- | --- |
|  |  | **CTR (n=10)** | |  | **SCC (n=10)** | |  | **ADENO (n=10)** | |  | **SCLC (n=8)** | |  |  | **CTR (n=8)** | |  | **SCC (n=4)** | |  | **ADENO (n=4)** | |  | **SCLC (n=8)** | |
| **Age ± SD** |  | 63 ± 5 | |  | 63 ± 5 | |  | 63 ± 7 | |  | 62 ± 8 | |  |  | 63 ± 6 | |  | 61 ± 6 | |  | 63 ± 7 | |  | 64 ± 6 | |
| **Sex** | **F** | 4 | 40% |  | 4 | 40% |  | 6 | 60% |  | 4 | 50% |  |  | 4 | 50% |  | 2 | 50% |  | 2 | 50% |  | 4 | 50% |
|  | **M** | 6 | 60% |  | 6 | 60% |  | 4 | 40% |  | 4 | 50% |  |  | 4 | 50% |  | 2 | 50% |  | 2 | 50% |  | 4 | 50% |
| **Race** | **B** | 0 | 0% |  | 3 | 30% |  | 5 | 50% |  | 3 | 38% |  |  | 2 | 25% |  | 3 | 75% |  | 2 | 50% |  | 4 | 50% |
|  | **W** | 10 | 100% |  | 7 | 70% |  | 5 | 50% |  | 5 | 62% |  |  | 6 | 75% |  | 1 | 25% |  | 2 | 50% |  | 4 | 50% |
| **Smoking status** | **Never smoked** | 0 | 0% |  | 0 | 0% |  | 0 | 0% |  | 0 | 0% |  |  | 0 | 0% |  | 0 | 0% |  | 0 | 0% |  | 0 | 0% |
|  | **Former smoker** | 3 | 30% |  | 5 | 50% |  | 5 | 50% |  | 3 | 38% |  |  | 5 | 62% |  | 1 | 25% |  | 4 | 100% |  | 2 | 25% |
|  | **Current smoker** | 7 | 70% |  | 5 | 50% |  | 5 | 50% |  | 5 | 62% |  |  | 3 | 38% |  | 3 | 75% |  | 0 | 0% |  | 6 | 75% |
| **Pk-Yr Hx ± SD** |  | 47 ± 18 | |  | 46 ± 25 | |  | 40 ± 23 | |  | 37 ± 18 | |  |  | 46 ± 18 | |  | 33 ± 5 | |  | 42 ± 31 | |  | 40 ± 27 | |
| **TNM** | **I-II** |  |  |  | 3 | 30% |  | 2 | 20% |  | 1 | 13% |  |  |  |  |  | 2 | 50% |  | 2 | 50% |  | 2 | 25% |
|  | **III** |  |  |  | 1 | 10% |  | 3 | 30% |  | 1 | 13% |  |  |  |  |  | 1 | 25% |  | 1 | 25% |  | 2 | 25% |
|  | **IV** |  |  |  | 6 | 60% |  | 5 | 50% |  | 6 | 74% |  |  |  |  |  | 1 | 25% |  | 1 | 25% |  | 4 | 50% |

**Supplementary Table 2.** NGS FC (fold change), P-value and FDR (false discovery rate) of miR-375 in the histological comparisons.

|  | **Entity_ID** | **P-Value** | **FDR** | **logFC** | **LinearFC** |
| --- | --- | --- | --- | --- | --- |
| SCLC vs CTR | hsa-miR-375 | 2.51227090E-07 | 0.000072353402 | 4.85394230 | 28.9189306 |
| SCLC vs SCC | hsa-miR-375 | 1.99329644E-07 | 0.0000568089486 | 5.02199615 | 32.4916286 |
| SCLC vs ADENO | hsa-miR-375 | 0.0358597530 | 0.95587457 | 2.10084773 | 4.28961371 |
| SCLC vs NSCLC | hsa-miR-375 | 0.00111775767 | 0.325267483 | 2.88616924 | 7.39304781 |

**Supplementary Table 3.** Performance of the classifier with number of true positive and true negative patients for each comparison.

|  |  | **Outcome +** | **Outcome -** | **Total** |
| --- | --- | --- | --- | --- |
| **SCLC vs CTR** | Test + | 5 | 2 | 7 |
|  | Test - | 11 | 15 | 26 |
|  | Total | 16 | 17 | 33 |
| **SCLC vs ADENO** | Test + | 6 | 0 | 6 |
|  | Test - | 10 | 7 | 17 |
|  | Total | 16 | 7 | 23 |
| **SCLC vs SCC** | Test + | 6 | 0 | 6 |
|  | Test - | 10 | 6 | 16 |
|  | Total | 16 | 6 | 22 |
| **SCLC vs NSCLC** | Test + | 6 | 0 | 6 |
|  | Test - | 10 | 13 | 23 |
|  | Total | 16 | 13 | 29 |
| **SCLC vs OTHER** | Test + | 5 | 0 | 5 |
|  | Test - | 11 | 30 | 41 |
|  | Total | 16 | 30 | 46 |
| **SCLC stage I+II vs CTR** | Test + | 2 | 8 | 10 |
|  | Test - | 2 | 9 | 11 |
|  | Total | 4 | 17 | 21 |
| **SCLC stage I+II+III vs CTR** | Test + | 4 | 10 | 14 |
|  | Test - | 5 | 7 | 12 |
|  | Total | 9 | 17 | 26 |
| **SCLC stage IV vs CTR** | Test + | 2 | 1 | 3 |
|  | Test - | 5 | 16 | 21 |
|  | Total | 7 | 17 | 24 |

## Supplementary File Legends

**Supplementary File 1.** Results from the sRNA seq data. The table sheets show the results obtained for each differential expression analysis performed: CTRL (control samples) Vs. SCLC (small cell lung cancer samples); SCC (lung squamous cell carcinoma samples) Vs. SCLC; ADENO (lung adenocarcinoma samples) Vs. SCLC; NSLC (non-small cell lung cancer samples) Vs. SCLC. For each of these analyses, there are two sheets: one for all expressed miRNAs analyzed and one for the significant ones considered in the downstream analyses. For the latter, there are two tables: the first on top shows the significant miRNAs filtered by the pvalue (pvalue<0.05), and the one on the bottom shows the significant miRNAs (pvalue<0.05) filtered by linear FC (|Linear FC|>1.5). In each table is reported the Entity ID (the name of the miRNA), Pvalue, FDR, Log Fold-change, Linear Fold-Change, the average and standard deviation of the expression of each miRNA for each condition, and the normalized expression of the miRNA along with the samples.

**Supplementary File 2.** Results from the NGS data. The table sheet named “All miRs” shows the results obtained for each differential expression analyses performed: Kruskal-Wallis omnibus test across all groups, and Wilcoxon Rank Sum test for BENIGN (control samples) vs. ADENO; SCLC vs. ADENO; Squam (lung squamous cell carcinoma samples) vs ADENO; BENIGN Vs. SCLC; BENIGN vs. Squam; Squam vs. SCLC. The table reports the name of the miRNA, Kruskal Wallis raw P-value and FDR, and Wicoxon Rank Sums raw P-value for each comparison.

The table sheets “SCLC_vs_AllElse_Results” and “SCLC_vs_AdenoSCC_Results” report the mean counts for each group and the raw P-values (Wicoxon Rank Sums) for the corresponding comparisons.

The table sheet named “Correlation” reports the Spearman correlations between the expression of the miRNAs with the miR-375-3p in the NGS data.

**Supplementary File 3.** Results from the Nanostring data. The table sheets show the results obtained for each differential expression analysis performed: CTRL (control samples) Vs. SCLC (small cell lung cancer samples); SCC (lung squamous cell carcinoma samples) Vs. SCLC; ADENO (lung adenocarcinoma samples) Vs. SCLC; NSLC (non-small cell lung cancer samples) Vs. SCLC. For each of these analyses, there are two sheets: one for all expressed miRNAs analyzed and one for the significant ones considered in the downstream analyses. For the latter, there are two tables: the first on top shows the significant miRNAs filtered by the pvalue (pvalue<0.05), and the one on the bottom shows the significant miRNAs (pvalue<0.05) filtered by linear FC (|Linear FC|>1.25) and expression (Expression counts > 30). In each table is reported the miRNA (the name of the miRNA), the accession number (MIMAT id), Pvalue, adjusted Pvalue, Log Fold-change, Linear Fold-Change, the geometric mean, standard deviation, and interquartile region (IQR) of the expression of each miRNA for each condition and the normalized expression of the miRNA along with the samples.
